# Risk Factor Associated with Negative Spouse HIV Seroconversion among Sero-Different Couples: A Nested Case-Control Retrospective Survey Study in 30 Counties in Rural China

**DOI:** 10.1371/journal.pone.0164761

**Published:** 2016-10-14

**Authors:** Houlin Tang, Zunyou Wu, Yurong Mao, Javier Cepeda, Jamie Morano

**Affiliations:** 1 Division of Integration and Evaluation, National Center for AIDS/STD Control and Prevention, Chinese Center for Disease Control and Prevention, Beijing, China; 2 National Center for AIDS/STD Control and Prevention, Chinese Center for Disease Control and Prevention, Beijing, China; 3 Department of Epidemiology, Johns Hopkins Bloomberg School of Public Health, Baltimore, Maryland, United States of America; 4 Division of Infectious Diseases and International Medicine, University of South Florida Morsani College of Medicine, Tampa, Florida, United States of America; National and Kapodistrian University of Athens, GREECE

## Abstract

**Background:**

Antiretroviral therapy (ART) and condom use have been proven to reduce the risk of sexual transmission of human immunodeficiency virus (HIV) among HIV sero-different couples, but its full implementation remains a challenge. This study aims to assess HIV seroconversion rate of HIV-negative spouse and its associated risk factors among HIV sero-different couples in rural China.

**Methods:**

An open cohort of HIV sero-different couples enrolled in 30 counties in China between October 1, 2010, and September 30, 2012, and followed-up to December 31, 2012, was constructed retrospectively. A nested case-control study of risk factors of HIV seroconversion among sero-different couples was conducted in April and May of 2013, based on the open cohort. Sero-different couples with the HIV-negative spouse seroconverting at least 3 months after the previous negative diagnosis during cohort observation period were labeled as “case couples”. The “control couples” were selected randomly from the same cohort that did not have the HIV-negative spouse seroconversion during the same period. The “case couples” and “control couples” were matched on gender, age, and region of residence. Sexual behaviors among HIV sero-different couples before and after the index spouses notifying their HIV infection status to their HIV-negative spouses were collected via face-to-face interview. Univariate and multivariate logistic regression models were used to assess factors associated with HIV seroconversion among HIV sero-different couples.

**Results:**

Of 4481 HIV sero-different couples, a total of 53 seroconversions were observed within 5218 person-years of follow-up. The incidence rate was 1.02 (95%CI: 0.76–1.33) per 100 person-years. Forty “case couples” confirmed HIV-negative spouse seroconversions infected via marital sexual transmission, were matched to 80 “control couples”. Of the 120 couples, 81(67.5%) were receiving ART, and 70 (58.3%) reported consistently used condoms during intercourse after the index spouse was diagnosed HIV infection. Multivariate conditional logistic regression analysis showed that the desire to conceive a child (OR = 5.18, 95% CI: 1.19–22.58) significantly increased the odds of HIV seroconversion. Protective factors of spousal HIV seroconversion were currently receiving ART (OR = 0.09, 95% CI: 0.01–0.67) and consistent condom use (OR = 0.05, 95% CI: 0.01–0.28).

**Conclusions:**

Intention to conceive a child is the most important risk factor for HIV seroconversion among sero-different couples. Specific efforts on scientific use of ART to assist sero-different couples to achieve their wish to conceive a healthy child are needed to minimize the risk of HIV transmission.

## Introduction

Many studies showed that, before the highly actively antiretroviral therapy (HAART) was available, the HIV incidence rate among the HIV negative partner in sero-different couples was between 4.6 and 11.8 per 100 person-years, with negative wives had a much higher HIV seroconversion rate than negative husbands among discordant couples [1, 2]. A few studies have been conducted in China, the incidence rate of HIV-negative spouses in sero-different couples ranged from 2.1 to 4.0 per 100 person-years for index spouse without taking HAART and from 0.8 to 1.1 per 100 person-year for index spouse taking HAART [3–5]. Several behavioral and biological factors such as high HIV viral load, advanced HIV disease, sexually transmitted infections, inconsistent condom use, frequent sexual contact, and number of partners have been associated with an increased risk of HIV transmission among heterosexual sero-different couples[4–6]. Effective viral suppression through antiretroviral therapy (ART) has been associated with a significant reduction in infectivity, therefore, diminished risk of HIV transmission in several studies [7, 8]. Antiretrovirals have substantial promise for HIV-1 prevention, either as for HIV-1–infected persons to reduce infectiousness, or as pre-exposure prophylaxis (PrEP) for HIV-1–uninfected persons to reduce the possibility of infection with HIV-1 [9–11].

Strategies on reducing HIV transmission among sero-different couples are early diagnosis, early initiation of ART, consistent condom use, and male circumcision[12]. In recent years, early initiation of ART has been increasingly recognized as a very important strategy for reducing HIV transmission among sero-different couples [13–15]. The benefit of ART in preventing new HIV infections has been confirmed by the HIV Prevention Trials Network (HPTN) 052 study, which found that early initiation of ART results in a 96% reduction in sexual transmission of HIV in short term[16]. The study has just confirmed that the 5 to 10 years’ long term impact of early ART on reduction of HIV sexual transmission remained as high as 93%[17]. The PARTNER study has further demonstrated that among sero-different couples of heterosexual and men who have sex with men (MSM) in which HIV-positive partners was using suppressive ART and who reported condomless sex, no HIV seroconversion was observed after the HIV-positive partner achieved viral load below 200 copies/ml[18]. Conclusive evidence for HIV treatment as prevention (TasP) represents a major victory in the fight against HIV/AIDS. However, broad-scale implementation of this strategy is expected to be expensive and challenging [3, 4, 14]. HIV sero-different couples received ART combined with condom use are very important for prevention of HIV sexual transmission. A meta-analysis found that ART reduced HIV transmission in couples with imperfect condom use by as much as 67%, but in couples reporting “consistent condom use” simultaneous use of ART, the prevention level could increase to 99% (95%CI: 0.96,1.00) [19]. It can reduce the risk of HIV seroconversion to nearly zero [20, 21]. Two recent studies conducted in two different rural areas (Yunnan and Henan) in China found conflicting results. One result confirmed that ART was associated with a 66% reduction in HIV incidence among discordant couples[5], but another result showed that ART was not significantly associated with a decreased risk of seroconversion[22]. This study aimed to assess seroconversion rate of HIV-negative spouses and risk factors associated with HIV sexual transmission between HIV sero-different couples in rural China.

## Materials and Methods

### Study design and participants

In China,individuals who tested HIV positive are reported to the Chinese HIV/AIDS Comprehensive Response Information Management System (CRIMS) for receiving follow-up health care services.[23] Information about marital status is collected in the initial epidemiological investigation for people newly diagnosed HIV infection. Local center for disease control and prevention (CDC) staff follow up individuals who reported having a spouse or regular sex partner with whom they cohabit, and encourage their spouses to be tested for HIV, with repeat HIV tests recommended every 6 months for spouses who test HIV negative[23]. Patient’s follow-up records in CRIMS also contain information on demographic characteristics, contact information, sexual behaviors, drug use risk behaviors, transmission routes, CD4+ T-cell test results, and subsequent treatment outcomes.

For couples with one spouse infected with HIV and another not infected with HIV is called “HIV sero-different couples”. The spouse of couple being first diagnosed HIV infection is called “Index spouse” and the spouse being tested HIV negative is called “HIV-negative spouse” in this study. The HIV-negative spouse become HIV antibody seroconverted, or in other words newly infected with HIV, during the study period, is called “seroconverted spouse”. Thirty counties with the most reported cases of sero-different couples from Yunnan, Guangxi, Henan, and Xinjiang four provinces and autonomous regions, were representative of the HIV epidemic throughout China, were selected for the study. An open cohort of HIV sero-different couples who newly diagnosed between October 1, 2010 and September 30, 2012 were retrospectively identified from the CRIMS database. The cohort of HIV-negative spouses within the sero-different couples were observed for HIV seroconversion from January 1, 2011 to December 31, 2012 retrospectively. A total of 4,481 HIV sero-different couples were enrolled and followed-up in the open cohort study constructed retrospectively.

A matched nested case-control study assessing the HIV seroconverting determinants among HIV sero-different couples was conducted in April and May 2013, based on the retrospective open cohort. A “case couple” was defined as the HIV-negative spouse of a sero-different couple was newly diagnosed with HIV infection more than 3 months after their enrollment of the cohort. A “control couple” was defined as the HIV-negative spouse of a sero-different couple remained consistently HIV negative during the study period. Each “case couple” was matched with two randomly selected “control couples”, or controls, from the retrospective cohort of sero-different couples. Matching variables were gender, age (±3 years old), date of the recent HIV test (±3 months) of index spouse, and region (county) of residence. Inclusion criteria for the “case couples” were: a) having a recent newly HIV positive serological test result for the HIV-negative spouses during the cohort observation period, b) being between 18 and 49 years of age, c) residing in the selected counties of the study, and d) providing written informed consent. Inclusion criteria for the “control couples” were the same except a) having a recent HIV negative serological test result for the HIV-negative spouses during the cohort observation period, and the recent HIV test date was within 3 months of seroconversion of the matched “case couple”.

The criteria of ART initiation for HIV-positive partners were in accordance with China’s national guidelines that ART is initiated regardless of CD4+ T-cell level[16]. HIV-positive spouses who started ART more than 3 months during the follow-up period were classified as ART-experienced group. Those who started ART less than 3 months during the follow-up period were still classified as ART-naïve group, given the fact that it usually takes 16–24 weeks to achieve viral suppression (viral load decrease below detectable level) among patients who take ART[24, 25].

### Data and sample collection

The HIV-negative spouses of sero-different couples were followed-up retrospectively every 3–6 months for HIV testing at the local community health centers or county CDCs during the study period. The primary outcome was HIV seroconversion of the HIV-negative spouses of sero-different couples. We defined a seroconversion as an individual who had a negative HIV test at baseline enrolling the cohort and subsequently had a positive HIV test during follow-up. Time of seroconversion was estimated as the mid-point between the last HIV-negative test and the first HIV-positive test. Demographic characteristics included sex, age, education level, marital status, occupation, nationality, and route of transmission, stage of disease, ART status, viral load, and the baseline and last recorded CD4+ T-cell counts of the Index spouses of HIV sero-different couples were directly obtained retrospectively from the CRIMS.

Face to face interview of “case couples” and “control couples” were conducted in a private place by experienced professionals of National Center for AIDS/STD Control and Prevention (NCAIDS), using a study specific structured questionnaire in Mandarin Chinese. Both spouses of each couple were asked about their demographic characteristics, sexual activities before and after seroconversion of the spouse, condom use, sexually transmitted infection (STI) history, intention to conceive a child, and awareness of prevention of HIV sexual transmission between sero-different couples. If HIV-negative spouses seroconverted during follow-up, they were asked to report sexual behaviors in the month(s) before their first HIV-positive test. A qualitative interview was also carried on with those couples.

### Statistical analysis

Categorical variables were presented in frequencies and percentages, and the Chi-square test (with Fisher’s exact test where appropriate) were used to compare demographic characteristics, sexual behavior, ART status, intention to conceive a child, drug use, alcohol use, and awareness of prevention of HIV sexual transmission in sero-different couples. HIV seroconversion rate was calculated by using the number of spouses who seroconverted as the numerator and the sum amount of at-risk time of all HIV-negative spouses in follow-up as the denominator. Those who did not seroconvert contributed follow-up time until the study end date. Those who had seroconverted during follow-up contributed their follow-up time until the time of seroconversion. Poisson 95% confidence intervals (CI) were calculated for overall seroconversion rate. Multi-categorical variables were transformed into dummy variables and then entered into a conditional logistic regression model. Univariate and multivariate conditional logistic regression models were used to assess the relationship between HIV seroconversion and the covariates. Demographic variables and other variables with p-values less than 0.1 from the univariate analysis were entered into a full multivariate conditional logistic regression model with step-wise selection used to include significant covariates. All hypothesis testing were based on 2-sided tests with alpha level of 0.05. Statistical analyses were performed by using SAS software (Version 9.3, SAS Institute, USA).

### Ethics statement

This study protocol, including design, recruitment, informed consent, was reviewed and approved by the Institutional Review Board of the National Center for AIDS/STD Control and Prevention, Chinese Center for Disease Control and Prevention (approval #X130205247). Written informed consent was obtained from all study participants.

## Results

### HIV seroconversions

**Fig 1:**
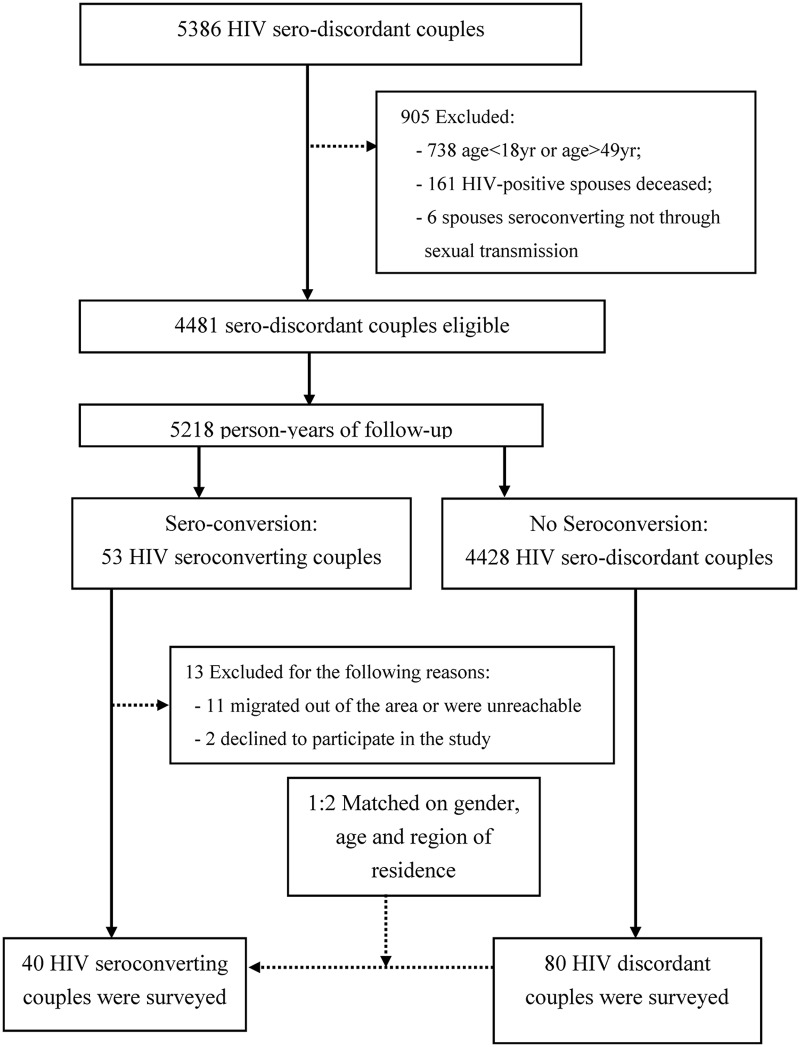
HIV Sero-different couple study eligibility flow diagram.

Of 4481 sero-different couples studied, 53 seroconversions were observed from January 1^st^, 2011 to December 31^st^, 2012 (Fig 1). The total follow-up time was 5218 person-years, and the HIV seroconversion rate was 1.02 per 100 person-years. A total of 40 seroconverting “case couples” were selected and 80 matched “control couples” were selected from the retrospective cohort for further study the risk factors for HIV seroconversion.

Of the 120 index partners, average age was 32.4±6.9 years, 75% were men, 32.5% were illiterate or only had completed primary school, 51.7% belonged to a minority, 59.2% were farmers, and 47.5% with total family income more than 30,000 Chinese Yuan (CYN) per year. Almost two-thirds of the entire study sample was living together for less than 1 year, but the vast majority (96.7%) was still living together with their spouses (Table 1).

**Table 1:** Baseline demographic comparison of characteristics of HIV sero-different couples based on index spouse, between case couples and control couples in China, 2011–2012.

Characteristics of Index Spouse	Case couples		Control couples		Total	
	N	%	N	%	N	%
Gender						
Male	30	75.0	60	75.0	90	75.0
Female	10	25.0	20	25.0	30	25.0
Education						
Illiteracy	6	15.0	2	2.5	8	6.7
Primary school	15	37.5	16	20.0	31	25.8
Middle school	16	40.0	43	53.8	59	49.2
High school	3	7.5	14	17.5	17	14.2
College	0	0.0	5	6.3	5	4.2
Ethnicity						
Han	21	52.5	37	46.3	58	48.3
Minority group	19	47.5	43	53.7	62	51.7
Occupation						
Farmer	29	72.5	42	52.5	71	59.2
Other	11	27.5	38	47.5	49	40.8
Family income (CYN)						
Less than 5,000	4	10.0	6	7.5	10	8.3
5,000–14,999	10	25.0	17	21.3	27	22.5
15,000–29,999	13	32.5	13	16.3	26	21.7
More than 30,000	13	32.5	44	55.0	57	47.5
Living with spouse						
≤ one year	23	57.5	48	60.0	71	59.2
> one year	17	42.5	32	40.0	49	40.8
Living apart						
Yes	1	2.5	3	3.8	4	3.3
No	39	97.5	77	96.3	116	96.7

Note: CYN = Chinese Yuan

### Sexual behaviors before index spouse diagnosed HIV infection

Of the 120 couples, 96 (80%) never used condoms when having sexual intercourse with spouses before the index spouse was diagnosed with HIV infection. This was significantly different between case couples (95.0%) and control couples (72.5%) (P = 0.03).

About one quarter of the index spouses had sex with casual partners outside of marriage before the index spouse was diagnosed with HIV infection, with 22.5% in case couples and 27.5% in control couples (P = 0.56). More than 80% never used condoms with casual partners however, the proportions were not significantly different between case couples and control couples (P = 0.50).

Almost one-third of the index spouses had sex with commercial partners before they were diagnosed with HIV, with 32.5% in case couples and 27.5% in control couples (P = 0.57). Of these, 88.6% never used condoms with commercial partners but this difference was not significant between case couples (84.6%) and control couples (90.9%) (P = 0.58) (Table 2).

**Table 2:** Sexual behavior characteristics of HIV sero-different couples before and after the index spouse diagnosed HIV infection in case couples and control couples in China, 2011–2012, N = 120.

Characteristics of index partner	Case couples		Control couples		χ^2^	P
	N = 40	%	N = 80	%		
Condom use between couples before the index spouse diagnosis of HIV infection						
Never	38	95.0	58	72.5	8.74*	0.03
Occasionally	2	5.0	14	17.5		
Usually	0	0.0	6	7.5		
Consistently	0	0.0	2	2.5		
Having extra marital partner before the index spouse diagnosis of HIV infection						
Yes	9	22.5	22	27.5	0.35	0.56
No	31	77.5	58	72.5		
Condom use with casual partners before the index spouse diagnosis of HIV infection						
Consistently	0	0.0	1	4.5	2.36*	0.50
Occasionally	0	0.0	4	18.2		
Never	9	100.0	17	77.3		
Having commercial sex partner before the index spouse diagnosis of HIV infection						
Yes	13	32.5	22	27.5	0.32	0.57
No	27	67.5	58	72.5		
Condom use with commercial partners before the index spouse diagnosis of HIV infection					0.31*	0.58
Consistently	0	0.0	0	0		
Occasionally	2	15.4	2	9.1		
Never	11	84.6	20	90.9		
Condom use between couples after the index spouse diagnosis of HIV infection						
Never	11	27.5	1	1.3	90.01*	<0.01
Occasionally	18	45	1	1.3		
Usually	11	27.5	8	10		
Consistently	0	0	70	87.5		
Reasons for condom use						
Prevention of HIV	28	96.6	79	100	5.05*	0.08
Other	1	3.4	0	0		
Frequency of sexual intercourse after the index spouse diagnosis of HIV infection						
Reduced	19	47.5	79	95	36.18	<0.01
Unchanged	21	52.5	4	5		
Frequency of sexual intercourse between couples in the past 6 months						
None	2	5	5	6.2	2.08	0.56
1–5 times	5	12.5	16	20		
6–9 times	14	35	31	38.8		
> = 10 times	19	47.5	28	35		
Condom use when having sex between couples in the past 6 months						
Never	12	31.6	0	0	58.9	<0.01
Occasionally	6	15.8	4	5.3		
Usually	12	31.6	3	4		
Consistently	8	21.1	68	90.7		
Having casual partners after the index spouse diagnosis of HIV infection						
Yes	1	2.5	1	1.2	0.25*	0.62
No	39	97.5	79	98.8		
Having commercial sex partner after the index spouse diagnosis of HIV infection						
Yes	1	2.5	0	0	2.00*	0.16
No	39	97.5	80	100		

*χ^2^ test was used with Fisher’ exact test.

One of HIV-negative spouse reported having sex with casual partners before the index spouse was diagnosed with HIV in the case group (data not showed in the table).

### Sexual behaviors after index spouse diagnosed HIV infection

Of the 120 couples, Table 2, 70 (58.3%) reported had consistently used condoms during intercourse with spouse after the index spouse was diagnosed HIV infection, with a proportion of 0% in the case couples and 87.5% in the control couples (P<0.01). The main reasons for not having consistently used condoms among seroconverted couples were as follows: hope to conceive a child (37.5%), the HIV-negative spouse thought no need to use (17.5%), both spouses thought no need to use (15.0%), forgot to use (12.5%), and unwilling to use (7.5%). Of the same respondents, 95 (79.2%) reported having less frequency of sexual intercourse after their index spouses diagnosed HIV infections, with 47.5% in the case couples and 95.0% in the control couples (P<0.01). In the past 6 months after index spouses diagnosis of HIV infection, 73 (60.8%) reported having sexual intercourse less than 10 times with 52.5% in the case couples and 65.0% in the control couples (P = 0.56). Only 12 (10.6%) in the case couples, while zero (0%) in the control couples reported having sexual intercourse with their spouse never used a condom.

Only 2 index spouses, one each in case couples and control couples, reported having sex with casual partners after the index spouse was diagnosed HIV infection. One index spouse in the case couples reported having sex with commercial sexual partners after the index spouse was diagnosed HIV infection, Table 2.

None of HIV-negative spouse reported having sex with casual partners or commercial partners after the index spouse was diagnosed with HIV infection (data was not showed in the table).

### Other HIV infected risk behaviors

Of the 120 index partners, 19 (15.8%) reported ever having a symptom suggestive of a sexually transmitted infection (STI), with 30.0% (12/40) in the case couples and 8.8% (7/80) in the control couples (P<0.01). Also, 69 (57.5%) reported ever had used recreational drugs with 52.5% (21/40) in the case couples and 60.0% (48/80) in the control couples. Of these, 17(14.2%) reported still using recreational drugs after their diagnosis of HIV infection with 25.0% (10/40) in the case couples and 8.8% (7/80) in the control couples (P = 0.02).

For alcohol use, 47 (39.2%) index spouses reported ever having alcoholic intemperance with 52.5% in the case couples and 32.5% in the control couples (P = 0.03). Of these, 21 (17.5%) index spouses reported still having alcohol intemperance after diagnosis HIV infection with 40.0% (16/40) in the case couples and 6.3% (5/80) in the control couples (P = 0.02).

### Stage of disease and CD4+ T cell count

Of the 120 couples, 53(44.2%) were AIDS patients, 35.0% in the case couples and 48.7% in the control couples (P = 0.15). Eighty-eight (73.3%) had their initial baseline CD4 +T-cell count less than 500 cells/mm^3^, with 87.5% in the case couples and 66.2% in the control couples (P = 0.05). Seventy-six (63.3%) had their most recent CD4 +T-cell count less than 500 cells/mm^3^, with 72.5% in the case couples and 58.5% in the control couples (P<0.05).

### Receiving ART in the case and control groups

Of the 120 couples, 81(67.5%) index spouses were receiving ART, with 35.0% (14/40) in the case couples and 83.8% (67/80) in the control couples (P<0.01). The main reasons for not receiving ART in 26 seroconverted couples were as follows: feeling well (34.6%), not receiving ART referral before seroconverting (34.6%), inconvenience to receive ART (7.7%), unwillingness to receive ART (7.7%), perceived ineligible for receiving ART (7.7%), worrying about side-effects (3.8%), and worrying about stigma and discrimination (3.8%). The main reasons for not receiving ART in 13 control couples as follows: feel well (38.5%), not receiving ART referral (38.5%), worry about side-effect (7.7%) and worry about stigma and discrimination (7.7%). Among 81 index spouses who received ART, 57 had their viral load testing results. The proportion of viral suppression was 57.1% (4/7) in case couples and 96% (48/50) in control couples (p = 0.011).

### Family support

Of the 120 couples, 117 (97.5%) index spouses reported that they had notified their spouses of HIV infections. Seventy percent of spouse notifications were carried out with assistance from doctors. Ninety-three (79.5%) reported that they notified HIV infection to their spouses within one month of their diagnose HIV infections. The proportions were 67.6% in the case couples and 85% in the control couples. Of 120 couples, 112 (95.7%) index spouses reported that their HIV-negative spouses expressed concerns and care about their health status and would support them.

Among 120 couples, 101(84.2%) reported having children. Of 120 index spouses, 32 (26.7%) reported to be willing to conceive a child in near future, with 40.0% (16/40) in the case couples and 20.0% (16/80) in the control couples (p = 0.02). Of 120 HIV-negative spouses, 45, including 24 HIV seroconverted during the study period and 21 remained HIV-negative, 37.5% (45/120) reported to be willing to have one child in near future, with 60.0% (24/40) in the case couples and 26.3% (21/80) in the control couples (p<0.01).

### Factors associated with HIV seroconversion

#### Univariate analysis

In the univariate conditional logistic regression model, factors associated with seroconversion among HIV sero-different couples were education level with primary school or below (OR = 5.00, 95% CI: 1.82–13.73, P<0.01), employment as a farmer or rural laborer (OR = 14.75, 95% CI: 1.86–117.09, P = 0.01), consistent condom use after diagnosis of HIV infection of the index partner (OR = 0.03, 95% CI: 0.01–0.15, P<0.01), using recreational drugs after diagnosis of HIV infection of the index spouse (OR = 5.09, 95% CI: 1.35–19.15, P = 0.02), alcohol use after diagnosis of HIV infection of the index spouse (OR = 12.19, 95% CI: 2.82–52.63, P<0.01), CD4+ T-cell count CD4<500 cells/mm^3^ at baseline (OR = 3.21, 95% CI: 1.17–8.83, P = 0.02), On ART (OR = 0.06, 95% CI: 0.02–0.20, P<0.01), time interval between the diagnose of index spouse and partner notification within one month (OR = 0.23, 95% CI: 0.08–0.65, P<0.01), intention to conceive a child (OR = 4.48, 95% CI: 1.41–14.18, P<0.01), awareness of ART reducing HIV sexual transmission among couples (OR = 0.07, 95% CI: 0.01–0.50, P = 0.01), and awareness of ART reducing HIV mother-to-child transmission (OR = 0.10, 95% CI: 0.03–0.34, P<0.01) (Table 3).

**Table 3:** Univariate conditional logistic regression analysis of factors associated with HIV seroconversion among sero-different couples (n = 120), in China, 2011–2012.

Variables of index spouse	OR*	95%CI	*χ*^*2*^	*P*
Ethnicity				
Minority	1.00			
Han	2.22	0.54–9.20	1.2	0.27
Education				
Middle school or above	1.00			
Primary school or below	5.00	1.82–13.73	9.76	<0.01
Occupation				
Other	1.00			
Farmer or rural laborer	14.75	1.86–117.09	6.48	0.01
Family income (RMB)				
<15 000	1.00			
≥15 000	2.00	0.55–7.28	1.11	0.29
Living apart				
Yes	1.00			
No	1.50	0.16–14.42	0.12	0.73
Living with spouse				
≤1year	1.00			
>1year	0.85	0.33~2.22	0.11	0.75
Condom use after index spouse diagnosis HIV infection				
Not consistently	1.00			
Consistently	0.03	0.01–0.15	21.21	<0.01
Having casual partners after the index spouse diagnosis HIV infection				
No	1.00			
Yes	2.00	0.13–31.97	0.24	0.62
Drug use after the index spouse diagnosis HIV infection				
No	1.00			
Yes	5.09	1.35–19.15	5.79	0.02
Alcohol use after the index spouse diagnosis HIV infection				
No	1.00			
Yes	12.19	2.82~52.63	11.21	<0.01
Baseline CD4+T-cell count**				
≥500 cells/mm^3^	1.00			
<500 cells/mm^3^	3.21	1.17–8.83	5.1	0.02
Recent CD4+ T-cell count				
≥500 cells/mm^3^	1.00			
<500 cells/mm^3^	1.86	0.82–4.24	2.19	0.14
On ART of index spouse				
No	1.00			
Yes	0.06	0.02–0.20	25.05	<0.01
Time interval between the diagnose of index spouse and spouse notification				
>1 month	1.00			
≤1 month	0.23	0.08–0.65	7.66	<0.01
Having children currently				
None	1.00			
Have	0.39	0.12–1.25	2.49	0.11
Intention to conceive a child				
No	1.00			
Yes	4.48	1.41–14.18	6.49	0.01
Awareness of condom use reducing HIV sexual transmission among couples				
No	1.00			
Yes	0.25	0.02–2.76	1.28	0.26
Awareness of ART reducing HIV sexual transmission among couples				
No	1.00			
Yes	0.07	0.01–0.50	6.84	0.01
Awareness of ART reducing mother-to-child transmission				
No	1.00			
Yes	0.10	0.03–0.34	13.43	<0.01

* OR = Odds Ratio;

**Baseline CD4+T-cell count: The CD4+ T-cell count of the positive spouse (index spouse) when a sero-different couple was enrolled in the study.

#### Multivariate analysis

Multivariate conditional logistic regression analysis was used to identify factors that were independently associated with HIV seroconversion among HIV sero-different couples. Firstly, after introducing the variables of education, occupation, drug use after diagnosis, alcohol use after diagnosis, CD4+ T-cell count at baseline, ART status, time interval between diagnose of index spouse and partner notification, intention to conceive a child, awareness of ART reducing HIV sexual transmission among couples, and awareness of ART reducing HIV mother-to-child transmission into the model, the index spouse of HIV sero-different on ART was significantly associated with a lower HIV seroconversion rate, comparing with those index spouses not on ART (OR = 0.04, 95% CI: 0.01–0.19, p<0.01). Index spouses of HIV sero-different couples with CD4+ T-cell counts 500 cells/mm^3^ had a significantly higher rate of HIV seroconversion, compared to those with CD4+ T-cell counts ≥500 cells/mm^3^ (OR = 11.39, 95% CI: 2.06–62.98, p<0.01).

Secondly, when the variables that were potentially modified by consistently condom use among discordant couples, were also included in the regression model, we found that discordant couples who consistently used condoms had a significantly lower rate of HIV seroconversion, compared to those who didn’t consistently used condoms (OR = 0.04, 95% CI: 0.01–0.16, p<0.01). HIV sero-different couples with an intention to conceive a child had a significantly higher risk of HIV seroconversion compared to those without intention to conceive a child (OR = 5.18, 95% CI: 1.19–22.58, p = 0.03). However, the index spouses of HIV sero-different couples on ART were no longer significantly associated with a lower rate of HIV seroconversion.

Thirdly, according to previous study findings, the covariates being on ART, intention to conceive a child and consistent condom use after diagnosis may be significantly associated with HIV-negative spouse seroconversion, these covariates were included in the last fitted model. Sero-different couples whose index spouses were receiving ART had a significantly lower rate of HIV seroconversion compared to those whose index spouses were not receiving ART (OR = 0.09, 95% CI: 0.01–0.67, p = 0.02). Discordant couples who consistently used condoms had a significantly lower rate of HIV seroconversion comparing with those inconsistently used condoms (OR = 0.05, 95% CI: 0.01–0.28, p<0.01). We also found that the variable intention to conceive a child was no longer significantly associated with HIV-negative spouses’ seroconversions (OR = 4.76, 95% CI: 0.87–25.96, p = 0.07). This result suggests that the impact of intention to conceive a child on spouse’s seroconversion was meditated through changes in these other variables (Table 4).

**Table 4:** Factors associated with HIV seroconversion among sero-different couples based on multivariate conditional logistic regression analysis in China, 2011–2011 (N = 120).

Variables	COR (95%CI)	p-value	AOR-1* (95%CI)	p-value	AOR-2** (95%CI)	p-value	AOR-3*** (95%CI)	p-value
Education								
Middle school or above	1							
Primary school or below	5.00 (1.82–13.73)	<0.01						
Occupation								
Other	1							
Farmer or rural laborer	14.75 (1.86–117.09)	0.01						
Condom use after HIV diagnosis								
Not consistently	1							
Consistently	0.03 (0.01–0.15)	<0.01			0.04 (0.01–0.16)	<0.01	0.05 (0.01–0.28)	<0.01
Drug use after HIV diagnosis								
No	1							
Yes	5.09 (1.35–19.15)	0.02						
Alcohol use after HIV diagnosis								
No	1							
Yes	12.19 (2.82–52.63)	<0.01						
CD4+ T cell count at baseline								
≥500 cells/mm^3^	1		1					
<500 cells/mm^3^	3.21 (1.17–8.83)	0.02	11.39 (2.06–62.98)	<0.01				
On ART								
No	1		1					
Yes	0.06 (0.02–0.20)	<0.01	0.04 (0.01–0.19)	<0.01			0.09 (0.01–0.67)	<0.01
Time interval between diagnose of index spouse and spouse notification								
>1month	1							
≤1month	0.23 (0.08–0.65)	<0.01						
Having children								
None	1							
Have	0.39 (0.12–1.25)	0.11						
Intention to conceive a child								
No	1		1					
Yes	4.48 (1.41–14.18)	0.01	4.34 (0.90–20.85)	0.067	5.18 (1.19–22.58)	0.03	4.76 (0.87–25.96)	0.07
Awareness of ART reducing sexual transmission among couples								
No	1							
Yes	0.07(0.01–0.50)	0.01						
Awareness of ART reducing mother-to-child transmission								
No	1							
Yes	0.10 (0.03–0.34)	<0.01						

*Adjusted for significant covariates: education, occupation, drug use after diagnosis, alcohol use after diagnosis, CD4+ T-cell count at baseline, on ART, time of spouse notification, intention to conceive a child, awareness of ART reducing sexual transmission among couples, awareness of ART reducing mother-to-child transmission, but not adjusted for condom use after diagnosis.

**Adjusted for significant covariates: education, occupation, drug use after diagnosis, alcohol use after diagnosis, CD4+ T-cell count at baseline, on ART, time of spouse notification, intention to conceive a child, awareness of ART reducing sexual transmission among couples, awareness of ART reducing mother-to-child transmission, as well as the variable of condom use after diagnosis.

***Adjusted for significant covariates: education, occupation, drug use after diagnosis, alcohol use after diagnosis, CD4+ T-cell count at baseline, on ART, time of spouse notification, intention to conceive a child, condom use after diagnosis, awareness of ART reducing sexual transmission among couples, awareness of ART reducing mother-to-child transmission, but the covariates of on ART, fertility desires and condom use after diagnosis must be in the last fitted model.

ART = antiretroviral therapy, COR = Crude Odds Ratio, AOR = Adjusted Odds Ratio

## Discussion

This study assessed HIV seroconversion rate and risk factors for HIV transmission between HIV sero-different couples in the marital context in rural China. Both quantitative and qualitative methods were used. Our study findings have important implications for reducing HIV transmission among sero-different couples.

First, early HIV diagnosis and early partner notification were mainstays of prevention of HIV sexual transmission between sero-different couples in China. We found that newly diagnosed HIV sero-different couples with early disclosure of less than one month decreased sexual risk-taking behaviors and increased condom use with their spouses after the diagnosis of HIV infection in index spouses. Kumarasamy et al found that patients who did not disclose their HIV status and who did not use condoms were more likely to be in relationships in which their spouse seroconverted[26]. Indeed, the earlier HIV status was disclosed to spouses, the less HIV transmissions ensued as this led to earlier adoption of protection measure between sero-different couples [27]. Our study added evidence to the literature as it demonstrated that the odds of HIV negative partner seroconversion was 4.3 times greater when index spouses waited more than one month to disclose their HIV status to their partner versus disclosure less than one month’s time.

Second, this study confirmed that the intention to conceive a child imparted a highly significant adjusted odds of HIV transmission between sero-different couples. This is consistent with prior findings that the frequency of sexual intercourse was associated with probability of HIV infection [28]. In our study, we found that most sero-different couples reduced the frequency of sexual intercourse after the index spouse diagnosis of HIV infection. Once conception occurred, pregnancy was also associated with an increased risk of HIV seroconversion in sero-different couples [29–31]. A cohort study of HIV-1 discordant couples in Kisumu, Kenya found that HIV uninfected individuals who were in partnerships in which conception occurred had a 1.8-fold increased risk of HIV acquisition compared with couples who did not conceive[32, 33]. Sixty-five percent of HIV seroconversions occurred within 6 months of conception or the first 6 months of pregnancy through unprotected sexual intercourse[32]. It is estimated that 20–50% of HIV positive individuals worldwide hope to conceive children, however this desire will put the negative spouse at the risk of HIV acquisition in the absence of preventative measures [32, 34, 35]. Our study found that the proportion of those wishing to conceive children in seroconverting couples was 40.0% in the index spouse and 60.0% in seroconverted spouses. The discordant couples who desired to naturally conceive children were 5.18 times as likely to seroconvert than those who did not, likely due to the fact that condoms were not being used as consistently in that situation.

Third, socioeconomic factors such as social gender inequality, rural cultural norms, low education levels, and intrauterine device and sterilization were also thought to have contributed to our study findings that the awareness of condom use in married discordant couples was poor before HIV diagnosis [36, 37]. We found that very few used condoms and that some did not know that condoms can be used for contraception before HIV diagnosis. By contrast, couples who consistently used condoms were 0.05 times less likely to seroconvert than those who did not after adjusting for other factors. As condom use has been shown to be associated with a decreased risk of seroconversion in sero-different couples [20, 38], more education on condom use and importance is needed among those in rural based settings to prevent initial HIV infection.

Fourth, this study found that active ART can effectively reduce the risk of seroconversion in sero-different couples as reduced serum HIV viral load has been shown to prevent HIV transmission from the well-known “treatment as prevention studies” [16, 39–41]. Our study found that the odds of seroconversion in sero-different couples in which the HIV-positive partner was receiving ART was 0.094 times than in those not receiving ART. Importantly, desire to conceive children was no longer a risk factor of seroconversion in sero-different couples when we included ART in the multivariate conditional logistic model (P = 0.07). Therefore, HIV sero-different couples attaining natural pregnancy were exposed to a much lower risk of sexual transmission of HIV when the infected partner on ART had suppression of viremia. However, more work needs to be done as some sero-different couples still cannot get timely ART. Therefore, we should strengthen counselling on early ART, timely transfer sero-different couples to ART clinics, and simplify the ART initiation procedures.

Our study has several limitations. First, this retrospective nested case control study utilized data from existing registries, and entries such as CD4+ T-cell counts or HIV testing records, may have been incomplete or missing. However, we attempted numerous times to find original records from the county CDC or hospital to confirm all records with the data from CRIMS. Also, the extensive number of records included ensured that the sero-different couples in case couples and control couples were carefully screened to ensure the most accurate representation from the cohort. Second, the classification of sexual transmission as marital within sero-different couples was identified by reported sexual behavior and may have resulted in misclassification of seroconverting couples as sexual transmission if other risk factors may not have been disclosed by the participant. However, trained national staff administered in–depth interviews to participants and confirm original records with sexual behaviors from the CRIMS in order to reduce bias due to one’s sexual risk behavior. Third, sexual behaviors were collected by person to person interview retrospectively. Recall bias and "social desirability" bias may result in underreporting risk sexual behavior. However, this kind of bias should be similar in both “case couples” and “control couples”. Therefore, the direction of bias should be towards to the null, and will not affect the study findings. Fourth, we didn’t have the HIV gene sequencing data to further accurately assess whether the transmission occurred within or out-with the marital partnership. Finally, suppressive ART on eliminating HIV sexual transmission is through reducing viral load to undetectable level[18], while in our study, all HIV-positive ART naïve index spouses had no RNA test results and only a small proportion of HIV-positive ART experienced index spouses had RNA test results. Thus, we are unable to assess whether or not treatment failure of index spouses was the primary cause of HIV seroconversion among HIV-negative spouse.

## Conclusions

In conclusion, this study demonstrates that early HIV disclosure and early initiation of ART for HIV index spouse minimize HIV sexual transmission to the HIV-negative spouses. Though the desire to conceive a child proved to be a significant risk factor for HIV transmission as a proxy for unprotected intercourse, the protective effects of ART use with HIV viral load suppression in combination with consistent condom use proved to be stronger mitigating factors hindering transmission. Specific efforts are needed to assist HIV sero-different couples to have a healthy child without occurring HIV sexual transmission among spouses.
